# Supplementary material for: Synthetic control over polymorph formation in the d-band semiconductor system FeS2
Source: Chem Sci. 2021 Oct 5;12(41):13870–7. doi: 10.1039/d1sc03026d (PMC8549780; doi:10.1039/d1sc03026d)
Supplement: SC-012-D1SC03026D-s001 [file SC-012-D1SC03026D-s001.pdf]

Electronic Supplementary Material (ESI) for Chemical Science.
This journal is © The Royal Society of Chemistry 2021

Supporting Information

Synthetic control over polymorph formation in the d-band semiconducting system FeS_2

KeYuan Ma, [1] Robin Lefèvre, [1] Qingtian Li, [2,3] Jorge Lago, [1,4] Olivier Blacque, [1] Wanli Yang, [2] and Fabian O. von Rohr [*,1]

1 Department of Chemistry, University of Zurich, CH-8057 Zurich, Switzerland

2 Advanced Light Source, Lawrence Berkeley National Laboratory, Berkeley, California94720, United States

3 State Key Laboratory of Functional Materials for Informatics, Shanghai Institute of Microsystem and Information Technology, Chinese Academy of Sciences, Shanghai 200050, China

4 Department of Inorganic Chemistry, Univ. del Pais Vasco (UPV-EHU), 48080 Bilbao,Spain

Experimental Section

Synthesis of FeS_2 polycrystalline samples:

The polycrystalline FeS_2 were synthesized by reacting elemental sulfur powder (purity 99.999 %) and $FeCl_2$ solution (1M) under hydrothermal synthesis conditions. Reactions were performed with varying pH values between 0.6 and 4.0 and under different reaction temperatures between 190 to 250 °C. The $FeCl_2$ solutions were prepared by dissolving $FeCl_2 \cdot 4H_2O$ (purity 99.99 %) in Millipore water. The pH values of the solutions were adjusted by the appropriate amounts of NaOH (8M) or HCl (8M) solutions. The pH values of the solutions were measured using a pH meter (Mettler Toledo) at room temperature (23 °C). 10 mL of a 1M $FeCl_2$ solution with different pH values and 4 mmol sulfur powder, corresponding to 128 mg, were placed in a polytetrauoroethylene (PTFE) lined stainless steel autoclave with a total volume of 15 ml (S-Figure 1, 2). To prevent any undesired side reactions, inert N_2 gas was filled in the upper volume of the autoclave. The autoclave was sealed and maintained at a constant temperature between 190-250 °C for 24 hours. Afterwards, the autoclave was cooled to room temperature by removing it from the furnace. The resulting precipitates were separated from the solution by filtration and washed sequentially with distilled water, toluene, and ethanol. After drying under N2 gas at room temperature, the obtained products were collected for further characterization. More than 100 successful syntheses have been carried out in this fashion in order to obtain a comprehensive synthesis map of this system.

Synthesis of marcasite single crystals:

Marcasite single crystals were synthesized by a space-separated hydrothermal reaction, where H_2S and S are produced in-situ during the reaction. A steel autoclave with a PTFE liner of the volume of 15 mL was used. The PTFE liner contained 6 ml of a 1M sodium thiosulphate solution ($Na_2S_2O_3 \cdot 5H_2O$, >99.5 %). A smaller PTFE beaker with a volume of 3 mL containing a 2 ml of a 1 M $FeCl_2 \cdot 4H_2O$ solution, was placed inside the autoclave. The volume ratio of the ($Na_2S_2O_3 \cdot 5H_2O$) solution to the $FeCl_2 \cdot 4H_2O$ solution was set to 3:1 (S-Figure 3(a)). The autoclave was heated to a constant temperature between 170 to 240 °C for 1 day and cooled to room temperature by removing it from the furnace. Marcasite crystals formed above the surface of $FeCl_2$ solutions and coated on the walls of the PTFE beaker (SFigure 3(a)). The collected crystals were washed sequentially with distilled water, toluene,

and ethanol to allow maximum dissolution of excess of sulfur. Finally, the single crystals were dried under N_2 gas at room temperature for further characterization.

To obtain very large marcasite single crystals for physical properties measurement, we used a very large steel autoclave with a PTFE liner of the volume of 115 mL containing the $Na_2S_2O_3$ solution, and a smaller PTFE beaker with a volume of 15 mL containing the $FeCl_2$ solutions (S-Figure 4). Large amounts of very large marcasite single crystals up to centimeters can be obtained by reacting the space-separated solutions at 180 °C for 14 days, or 230 and 240 °C for 2-3 days (S-Figure 3,4). High quality pure marcasite single crystals were collected on the wall of the inner PTFE beaker above $FeCl_2$ solution surface as shown in the supporting information. Marcasite can transform into pyrite under high temperatures. Based on the obtained large marcasite single crystals, we used an annealing process to obtain large pyrite single crystals under argon atmosphere. We found the large marcasite single crystals can be transformed to pyrite between 350 °C and 500 °C in argon. Single crystal measurements on these pyrite crystals showed no difference with pyrite single crystals prepared by other methods (S-Table 5).

Characterization:

The crystal structure and phase purity of the samples were investigated by powder X-ray diffraction (PXRD) measurements on a STOE STADIP diffractometer with Mo Kα radiation (λ = 0.709300 Å). The PXRD patterns were collected in the 2-θ range of 10-40° and a scan rate of 0.25°/min. Rietveld refinements were performed using WinPlotr included in the package FullProf. Single-crystal X-ray diffraction data was collected at 160(1) K on a Rigaku OD XtaLAB Synergy, Dualex, Pilatus 200 K diffractometer using a single wavelength X-ray source (Mo Kα radiation: λ = 0.71073 Å)[1] from a micro-focus sealed X-ray tube and an Oxford liquid-nitrogen Cryostream cooler. The selected suitable single crystal was mounted using polybutene oil on a flexible loop fixed on a goniometer head and immediately transferred to the diffractometer. Pre-experiment, data collection, data reduction and analytical absorption correction[2] were performed with the program suite *CrysAlisPro*[3]. Using *Olex*2,[4] the structure was solved with the SHELXT[5] small molecule structure solution program and refined with the SHELXL 2018/3 program package[6] by full-matrix least-squares minimization on F^2. PLATON[7] was used to check the results of the X-ray analysis. The micro-morphology of marcasite was observed by a scan electron microscopy (SEM) coupled with energy-dispersive spectroscopy (EDS). In order to determine

the sulfur content in the marcasite crystals, thermogravimetric measurement was performed on a Netzsch Jupiter STA 449 F3 TGA in pure O_2 atmosphere with about 30 mg of marcasite single crystal placed in a small Al_2O_3 crucible at a temperature range of 25-800 °C. After measurement, the residual products were collected for PXRD measurement.

The electronic band gap was determined by means of X-ray absorption/emission spectroscopy (XAS/XES) measurements on the marcasite and pyrite single crystals. The measurements were performed on beamline 8.0.1.1 at the Advanced Light Source, Lawrence Berkeley National Laboratory. The X-ray absorption and emission spectra of marcasite and pyrite single crystals were recorded at the S L-edge. The optical band gap was determined from Tauc plots obtained from diffuse reflectance spectrum measured on a Shimadzu UV-vis-NIR 3600 spectrometer at a range of 200-2000 nm at room temperature. In the measurement, barium sulfate ($BaSO_4$) powder was used as the standard and background sample. Subsequently, marcasite and pyrite single crystals were grounded into powder in a mortar and spread on the barium sulfate background powder. Temperature dependent magnetization measurement was performed using a Quantum Design magnetic properties measurement system (MPMSXL) equipped with a reciprocating sample option (RSO). The Hall resistance and electric transport measurements were performed with a Quantum Design physical property measurement system (PPMS). The standard four-probe technique was employed to measure the electrical resistivity. In the resistivity measurement, gold wires were connected to the sample with silver paint.

1. Used polytetrafluoroethylene liners and beaker for reactions

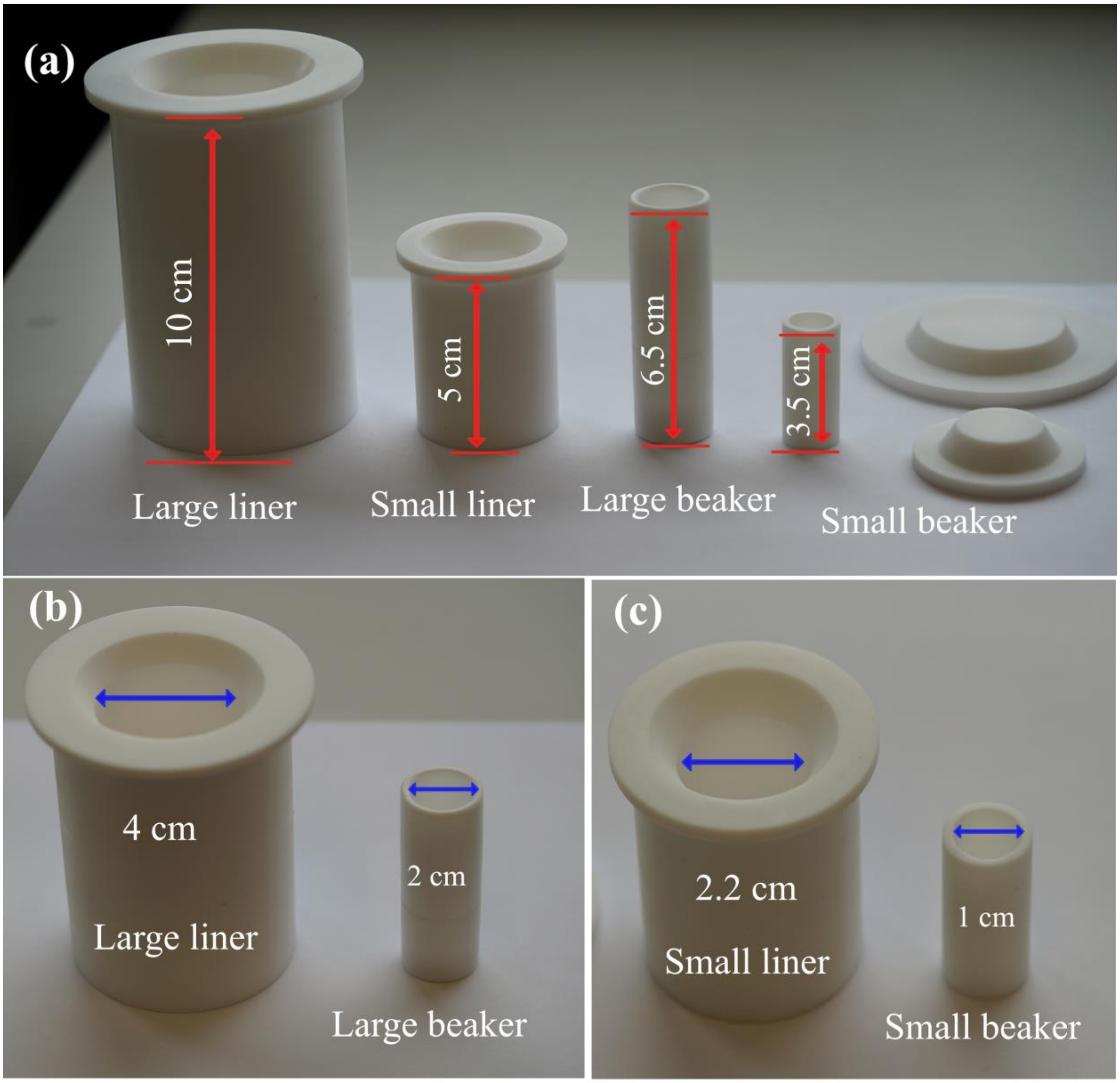

S-Figure1. Photographs of the polytetrafluoroethylene (PTFE) liners and beaker used for the hydrothermal synthesis reactions.

2. Synthesis of the FeS_2 polycrystalline samples:

The polycrystalline samples were prepared by directly reacting sulfur powder with $FeCl_2$ solutions under different pH values and temperatures.

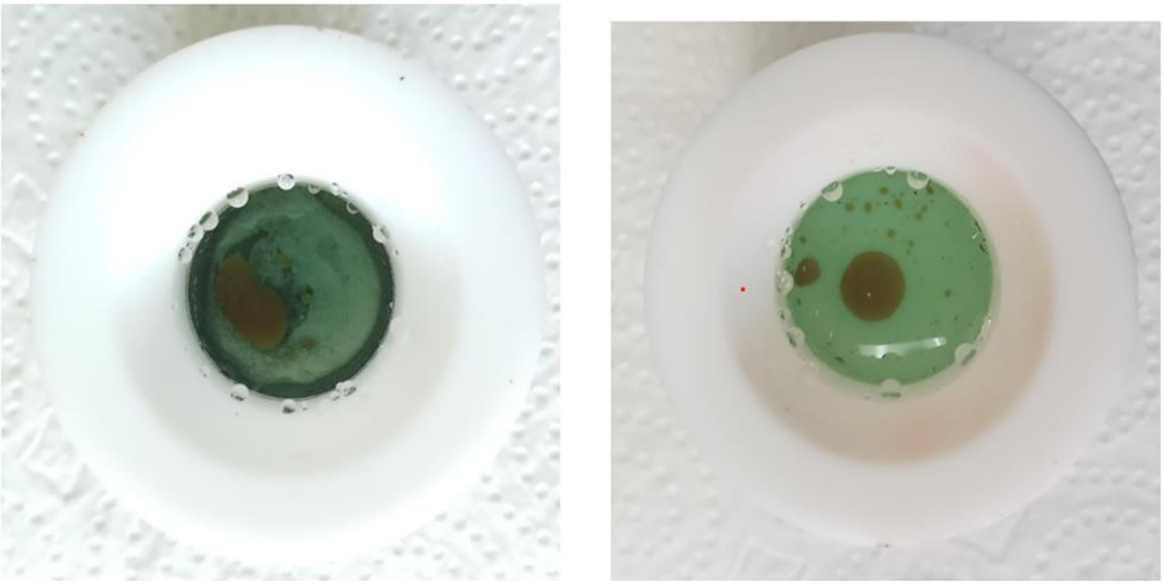

S-Figure2. $FeCl_2$-S reaction system: with FeS_2 products on the wall of the small PTFE liner (left). no FeS_2 products are formed due to the very low pH value (0.51) of the $FeCl_2$ solution (right). After reaction, the sulfur powder aggregated into large sulfur particles.

S-Table 2. Crystallographic data of polycrystalline FeS_2 marcasite and pyrite from Rietveld refinements.

**FeS_2 Marcasite**	
Space group: Pnnm (58)	Crystal System: Orthorhombic
Lattice parameters: a = 4.4478 Å, b = 5.4268 Å, c = 3.3852 Å.	
$\alpha = \beta = \gamma = 90°$	
Cell Volume: 81.7111 Å^3	Density: 4.876 g/cm^3
**FeS_2 Pyrite**	
Space group: Pa-3 (205).	Crystal System: Cubic
Lattice parameters: a = b = c = 5.4195Å	
$\alpha = \beta = \gamma = 90°$	
Cell Volume: 159.1780 Å^3	Density: 5.006 g/cm^3

3. PXRD refinement results for the FeCl2-S reaction system under different temperatures and pH values

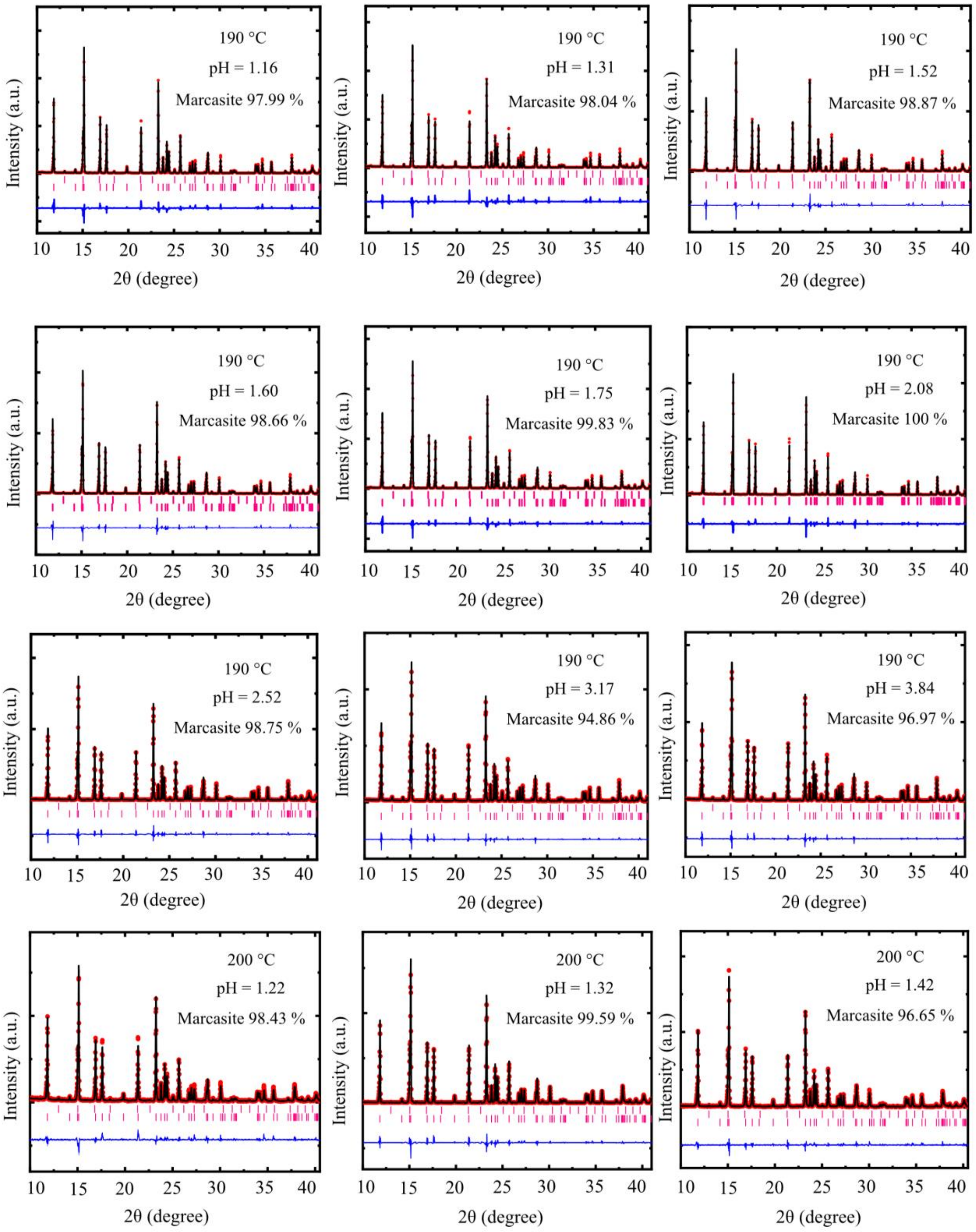
190 °C
pH = 1.16
Marcasite 97.99 %
190 °C
pH = 1.31
Marcasite 98.04 %
190 °C
pH = 1.52
Marcasite 98.87 %
190 °C
pH = 1.60
Marcasite 98.66 %
190 °C
pH = 1.75
Marcasite 99.83 %
190 °C
pH = 2.08
Marcasite 100 %
190 °C
pH = 2.52
Marcasite 98.75 %
190 °C
pH = 3.17
Marcasite 94.86 %
190 °C
pH = 3.84
Marcasite 96.97 %
200 °C
pH = 1.22
Marcasite 98.43 %
200 °C
pH = 1.32
Marcasite 99.59 %
200 °C
pH = 1.42
Marcasite 96.65 %
Intensity (a.u.)
2θ (degree)
10
15
20
25
30
35
40

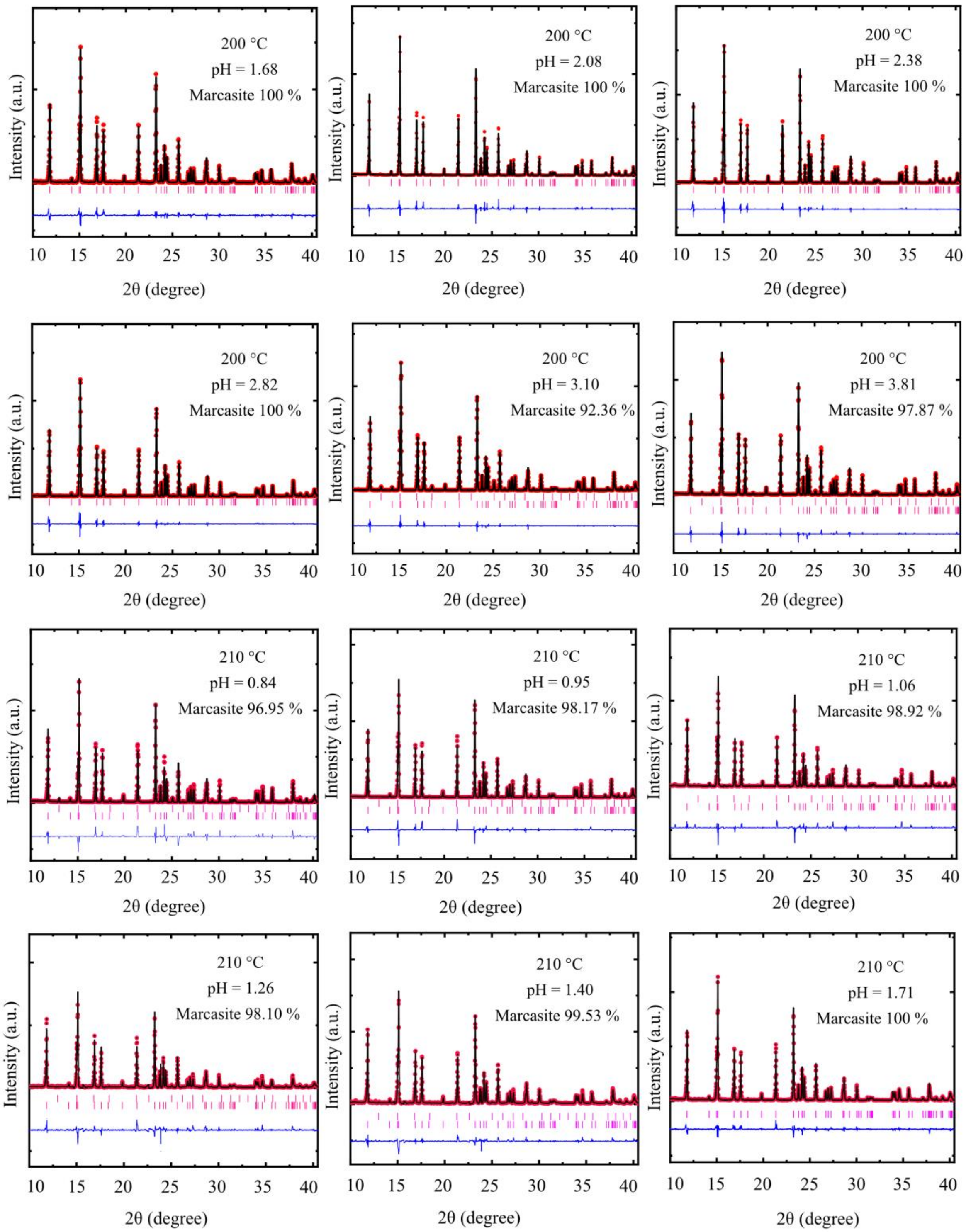
200 °C
pH = 1.68
Marcasite 100 %
200 °C
pH = 2.08
Marcasite 100 %
200 °C
pH = 2.38
Marcasite 100 %
200 °C
pH = 2.82
Marcasite 100 %
200 °C
pH = 3.10
Marcasite 92.36 %
200 °C
pH = 3.81
Marcasite 97.87 %
210 °C
pH = 0.84
Marcasite 96.95 %
210 °C
pH = 0.95
Marcasite 98.17 %
210 °C
pH = 1.06
Marcasite 98.92 %
210 °C
pH = 1.26
Marcasite 98.10 %
210 °C
pH = 1.40
Marcasite 99.53 %
210 °C
pH = 1.71
Marcasite 100 %
Intensity (a.u.)
2θ (degree)
10 15 20 25 30 35 40

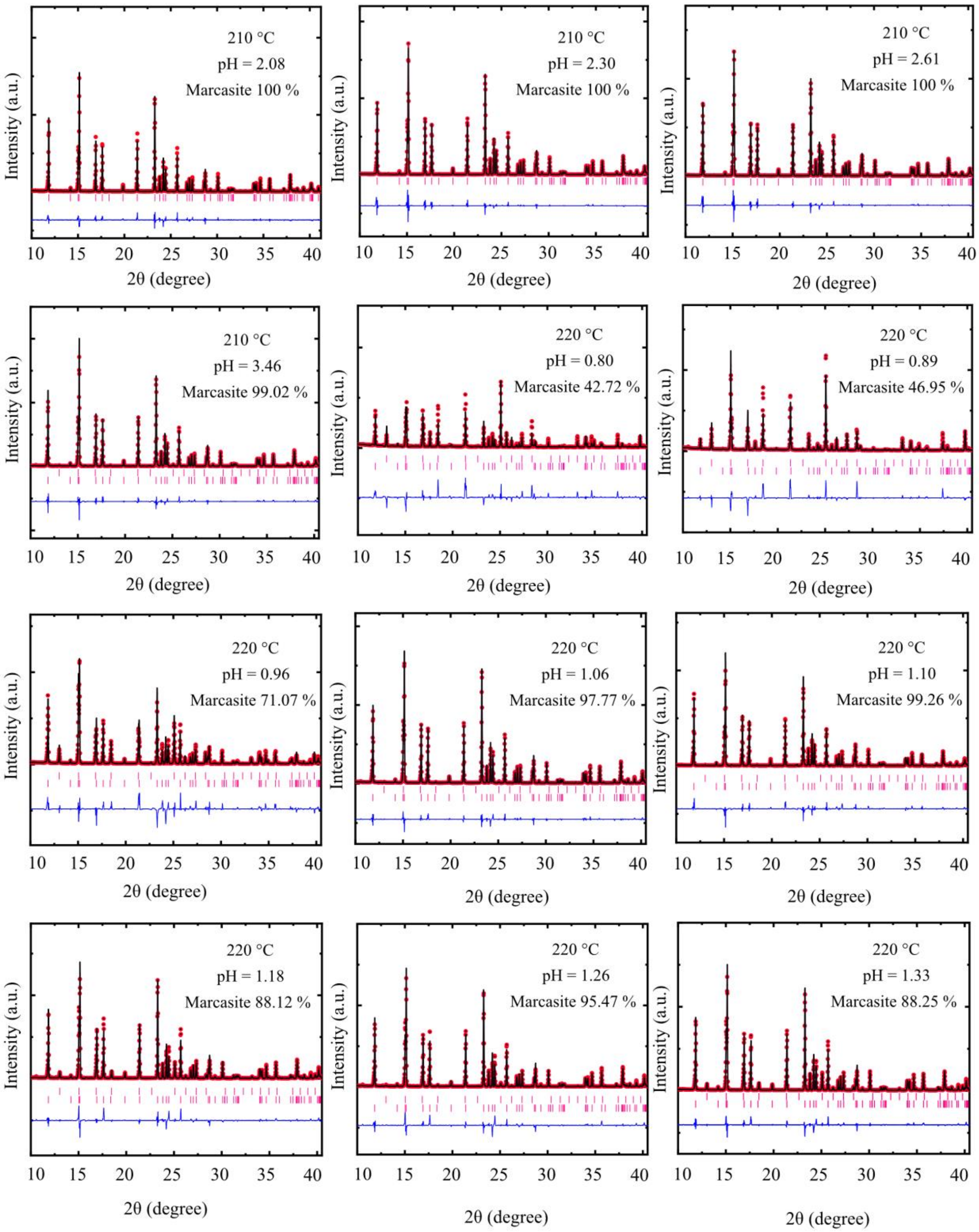
210 °C
pH = 2.08
Marcasite 100 %
210 °C
pH = 2.30
Marcasite 100 %
210 °C
pH = 2.61
Marcasite 100 %
210 °C
pH = 3.46
Marcasite 99.02 %
220 °C
pH = 0.80
Marcasite 42.72 %
220 °C
pH = 0.89
Marcasite 46.95 %
220 °C
pH = 0.96
Marcasite 71.07 %
220 °C
pH = 1.06
Marcasite 97.77 %
220 °C
pH = 1.10
Marcasite 99.26 %
220 °C
pH = 1.18
Marcasite 88.12 %
220 °C
pH = 1.26
Marcasite 95.47 %
220 °C
pH = 1.33
Marcasite 88.25 %
Intensity (a.u.)
2θ (degree)
10
15
20
25
30
35
40

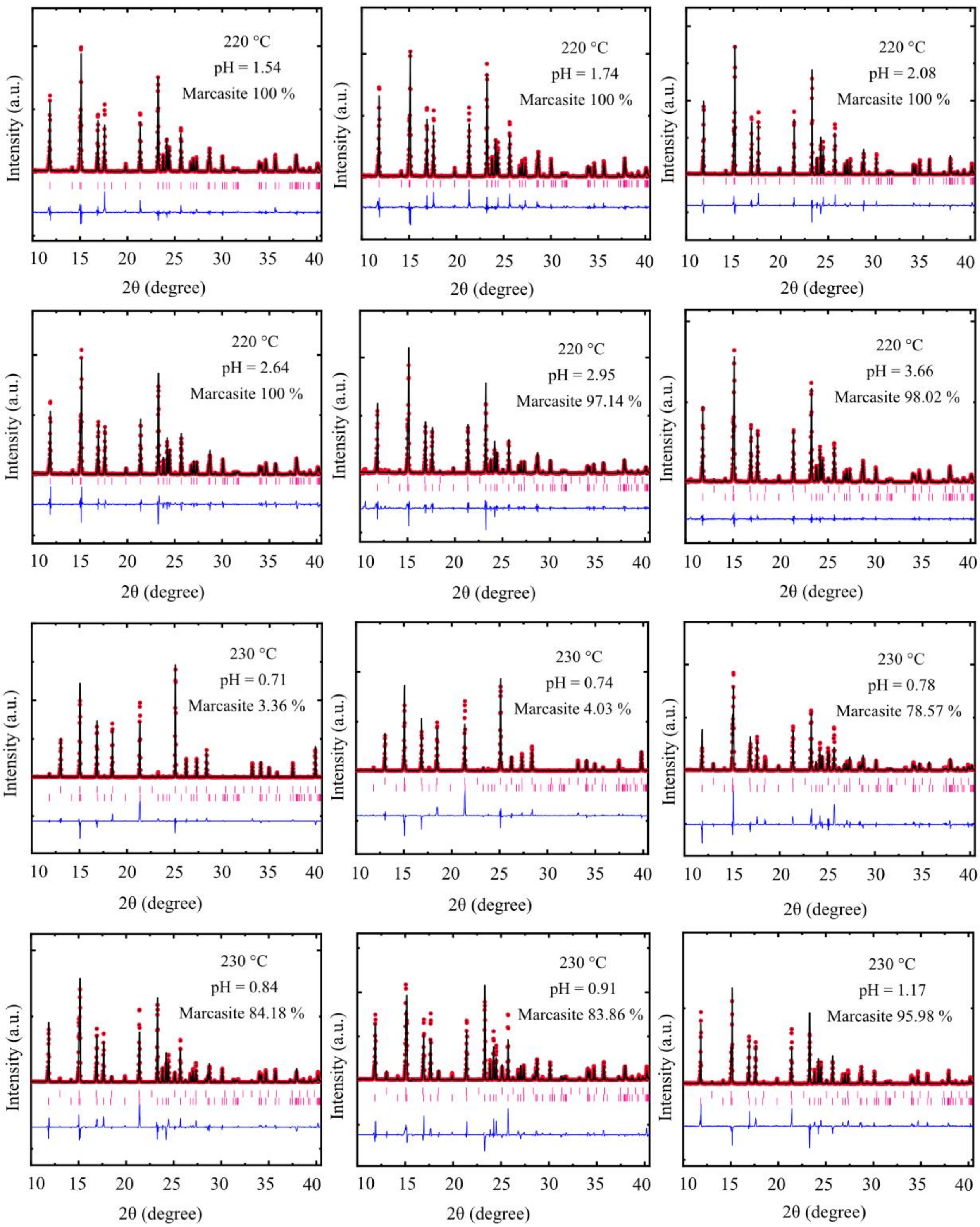
220 °C
pH = 1.54
Marcasite 100 %
220 °C
pH = 1.74
Marcasite 100 %
220 °C
pH = 2.08
Marcasite 100 %
220 °C
pH = 2.64
Marcasite 100 %
220 °C
pH = 2.95
Marcasite 97.14 %
220 °C
pH = 3.66
Marcasite 98.02 %
230 °C
pH = 0.71
Marcasite 3.36 %
230 °C
pH = 0.74
Marcasite 4.03 %
230 °C
pH = 0.78
Marcasite 78.57 %
230 °C
pH = 0.84
Marcasite 84.18 %
230 °C
pH = 0.91
Marcasite 83.86 %
230 °C
pH = 1.17
Marcasite 95.98 %
Intensity (a.u.)
2θ (degree)
10 15 20 25 30 35 40

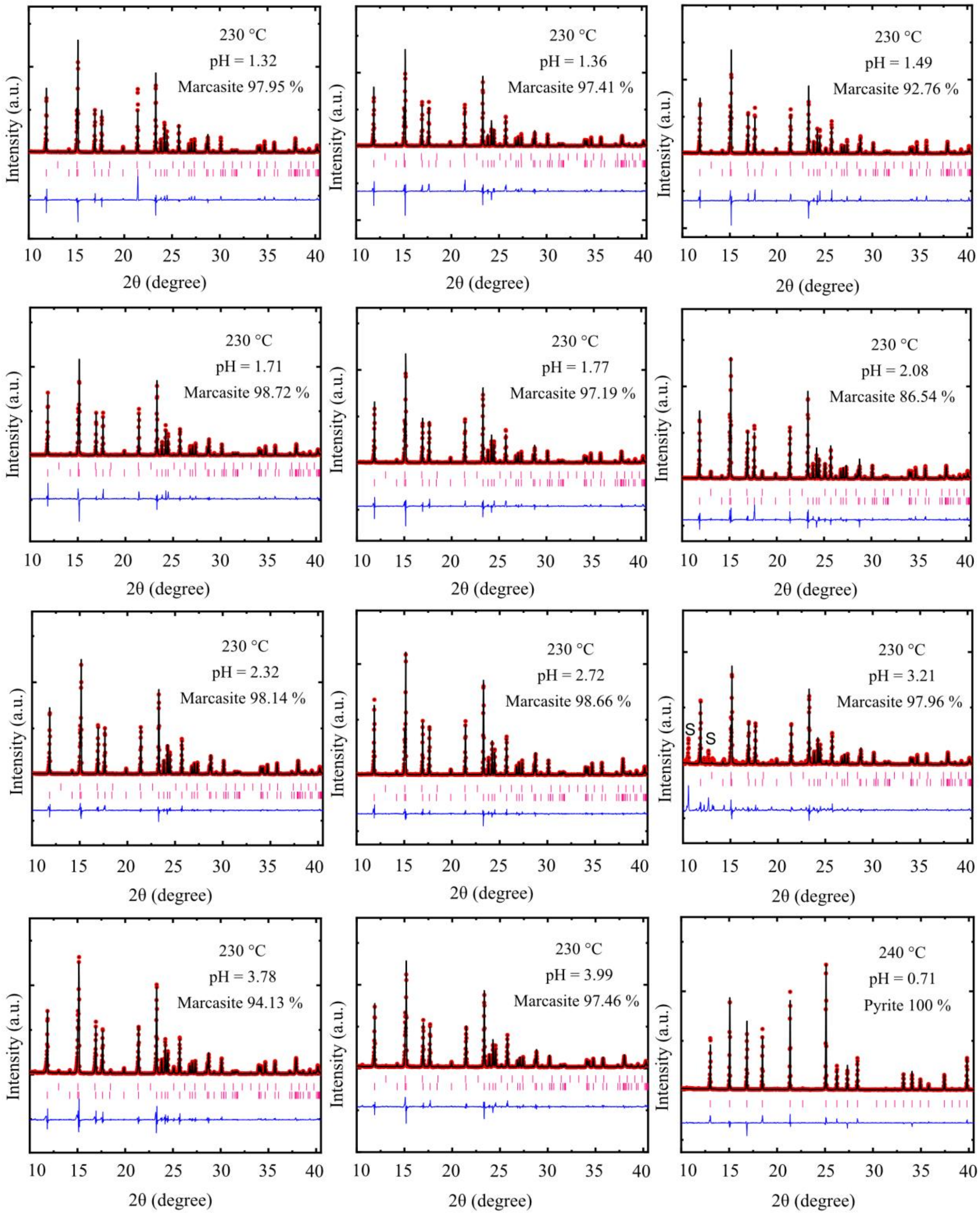
230 °C
pH = 1.32
Marcasite 97.95 %
230 °C
pH = 1.36
Marcasite 97.41 %
230 °C
pH = 1.49
Marcasite 92.76 %
230 °C
pH = 1.71
Marcasite 98.72 %
230 °C
pH = 1.77
Marcasite 97.19 %
230 °C
pH = 2.08
Marcasite 86.54 %
230 °C
pH = 2.32
Marcasite 98.14 %
230 °C
pH = 2.72
Marcasite 98.66 %
230 °C
pH = 3.21
Marcasite 97.96 %
S
S
230 °C
pH = 3.78
Marcasite 94.13 %
230 °C
pH = 3.99
Marcasite 97.46 %
240 °C
pH = 0.71
Pyrite 100 %
Intensity (a.u.)
2θ (degree)
10
15
20
25
30
35
40

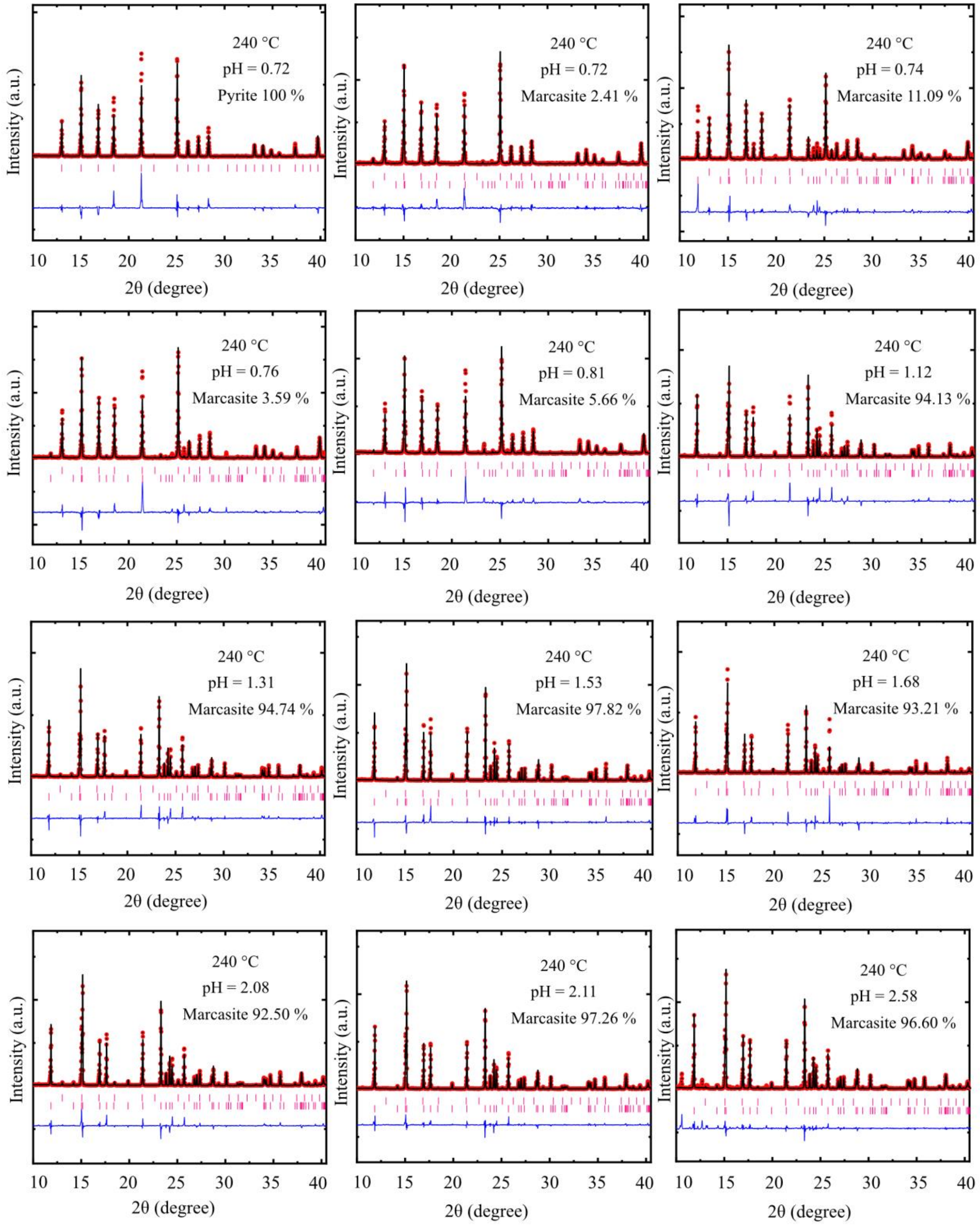

240 °C
pH = 0.72
Pyrite 100 %
240 °C
pH = 0.72
Marcasite 2.41 %
240 °C
pH = 0.74
Marcasite 11.09 %
240 °C
pH = 0.76
Marcasite 3.59 %
240 °C
pH = 0.81
Marcasite 5.66 %
240 °C
pH = 1.12
Marcasite 94.13 %
240 °C
pH = 1.31
Marcasite 94.74 %
240 °C
pH = 1.53
Marcasite 97.82 %
240 °C
pH = 1.68
Marcasite 93.21 %
240 °C
pH = 2.08
Marcasite 92.50 %
240 °C
pH = 2.11
Marcasite 97.26 %
240 °C
pH = 2.58
Marcasite 96.60 %
Intensity (a.u.)
2θ (degree)
10 15 20 25 30 35 40

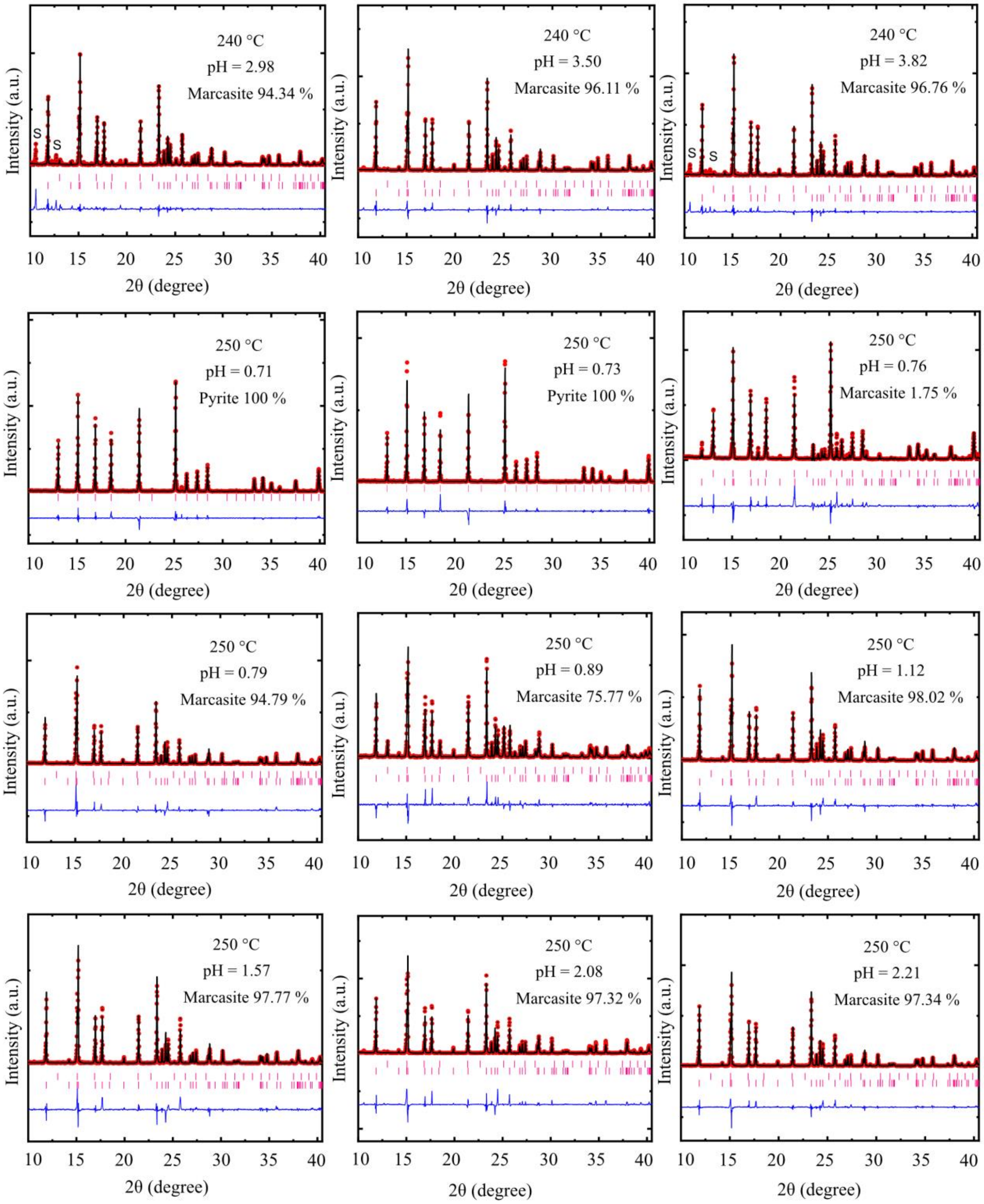
240 °C
pH = 2.98
Marcasite 94.34 %
240 °C
pH = 3.50
Marcasite 96.11 %
240 °C
pH = 3.82
Marcasite 96.76 %
250 °C
pH = 0.71
Pyrite 100 %
250 °C
pH = 0.73
Pyrite 100 %
250 °C
pH = 0.76
Marcasite 1.75 %
250 °C
pH = 0.79
Marcasite 94.79 %
250 °C
pH = 0.89
Marcasite 75.77 %
250 °C
pH = 1.12
Marcasite 98.02 %
250 °C
pH = 1.57
Marcasite 97.77 %
250 °C
pH = 2.08
Marcasite 97.32 %
250 °C
pH = 2.21
Marcasite 97.34 %
S
Intensity (a.u.)
2θ (degree)
10 15 20 25 30 35 40

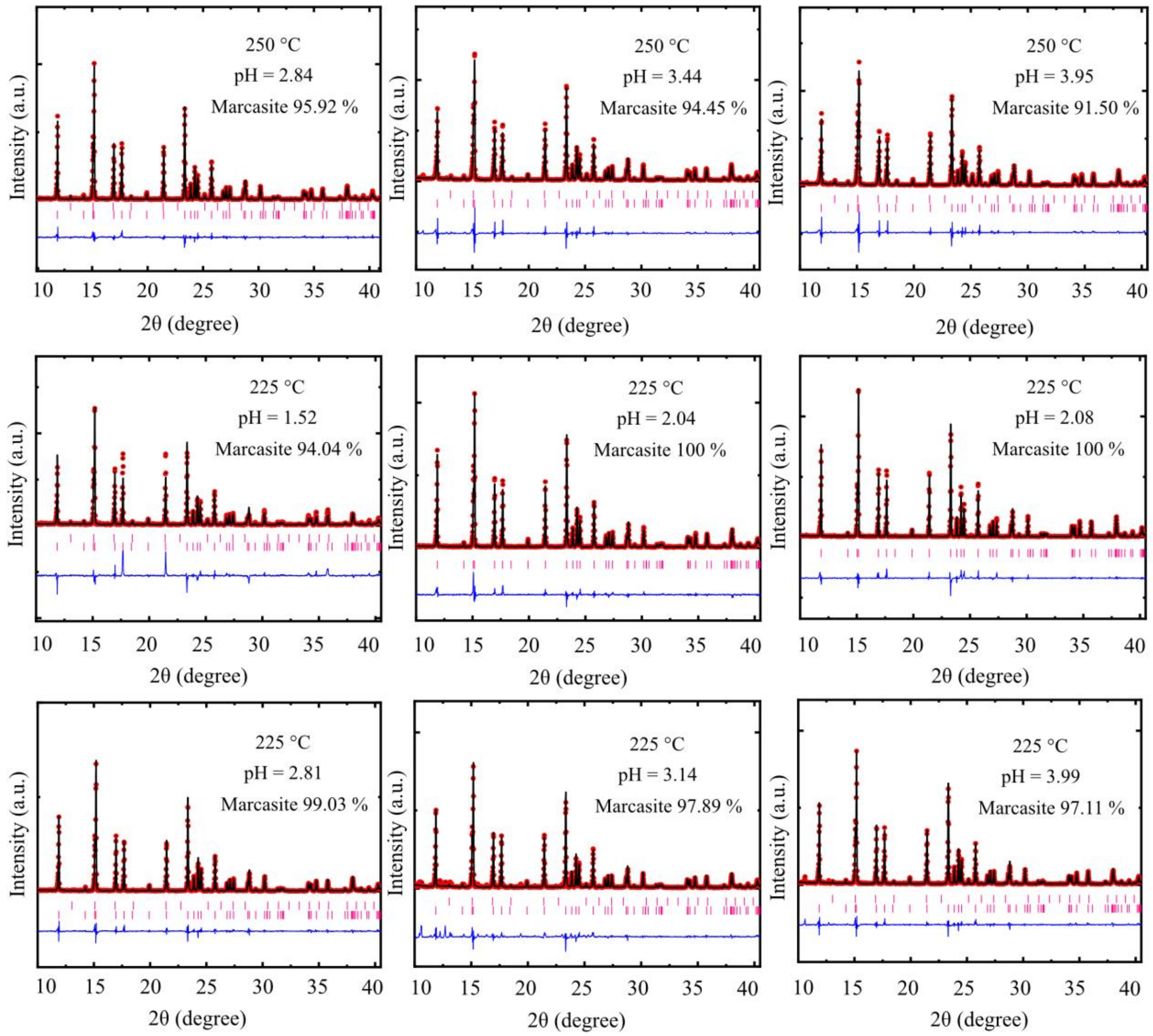
250 °C
pH = 2.84
Marcasite 95.92 %
250 °C
pH = 3.44
Marcasite 94.45 %
250 °C
pH = 3.95
Marcasite 91.50 %
225 °C
pH = 1.52
Marcasite 94.04 %
225 °C
pH = 2.04
Marcasite 100 %
225 °C
pH = 2.08
Marcasite 100 %
225 °C
pH = 2.81
Marcasite 99.03 %
225 °C
pH = 3.14
Marcasite 97.89 %
225 °C
pH = 3.99
Marcasite 97.11 %
Intensity (a.u.)
2θ (degree)

S-Table 3. PXRD refinement results for the $FeCl_2$-S reaction system under different temperatures and pH values

Number	pH value	Temperature (˚C)	percentage of Marcasite (%)
1	2.08	180	No FeS_2 products
2	2.08	185	No FeS_2 products
3	0.90	190	No FeS_2 products
4	1.10	190	No FeS_2 products
5	1.16	190	97.99
6	1.31	190	98.04
7	1.52	190	98.87
8	1.60	190	98.66
9	1.75	190	99.83
10	2.08	190	100
11	2.52	190	98.78
12	3.17	190	94.86
13	3.84	190	96.97
14	0.85	200	No FeS_2 products
15	0.96	200	No FeS_2 products
16	1.22	200	98.43
17	1.32	200	99.59
18	1.42	200	99.65
19	1.68	200	100
20	2.08	200	100
21	2.38	200	100
22	2.82	200	100
23	3.10	200	92.36
24	3.81	200	97.87

Number	pH value	Temperature (˚C)	percentage of Marcasite (%)
25	0.74	210	No FeS_2 products
26	0.76	210	No FeS_2 products
27	0.84	210	96.95
28	0.95	210	98.17
29	1.06	210	98.92
30	1.26	210	98.10
31	1.40	210	99.53
32	1.71	210	100
33	2.08	210	100
34	2.30	210	100
35	2.61	210	100
36	3.46	210	99.02
37	0.78	220	No FeS_2 products
38	0.80	220	42.72
39	0.89	220	46.95
40	0.96	220	71.97
41	1.10	220	99.26
42	1.06	220	97.77
43	1.18	220	88.12
44	1.26	220	95.47
45	1.33	220	88.25
46	1.74	220	100
47	2.08	220	100
48	2.64	220	100
49	2.95	220	97.14
50	3.66	220	98.02

Number	pH value	Temperature (˚C)	percentage of Marcasite (%)
51	0.60	230	No FeS_2 products
52	0.65	230	No FeS_2 products
53	0.71	230	3.36
54	0.74	230	4.03
55	0.76	230	72.61
56	0.78	230	78.57
57	0.84	230	84.18
58	0.91	230	83.86
59	1.17	230	95.98
60	1.32	230	97.95
61	1.36	230	97.41
62	1.49	230	92.76
63	1.71	230	98.72
64	1.77	230	97.19
65	2.08	230	86.54
66	2.32	230	98.14
67	2.72	230	98.66
68	3.21	230	97.96
69	3.78	230	94.13
70	3.99	230	97.46
71	0.65	240	No FeS_2 products
72	0.71	240	0
73	0.72	240	0
74	0.73	240	2.41
75	0.74	240	11.09

Number	pH value	Temperature (˚C)	percentage of Marcasite (%)
76	0.76	240	3.59
77	0.81	240	5.66
78	1.12	240	94.13
79	1.31	240	94.74
80	1.53	240	97.82
81	1.68	240	93.21
82	2.08	240	92.50
83	2.11	240	97.26
84	2.58	240	96.60
85	2.98	240	94.34
86	3.50	240	96.11
87	3.82	240	96.76
88	0.48	250	No FeS_2 products
89	0.54	250	No FeS_2 products
90	0.71	250	0
91	0.73	250	0
92	0.76	250	1.75
93	0.79	250	94.79
94	0.89	250	75.77
95	1.12	250	98.02
96	1.57	250	97.77
97	2.08	250	97.32
98	2.21	250	97.34
99	2.84	250	95.92
100	3.44	250	94.45
101	3.95	250	91.50

4. Synthesis of FeS_2 Marcasite single crystals with space separated reaction:

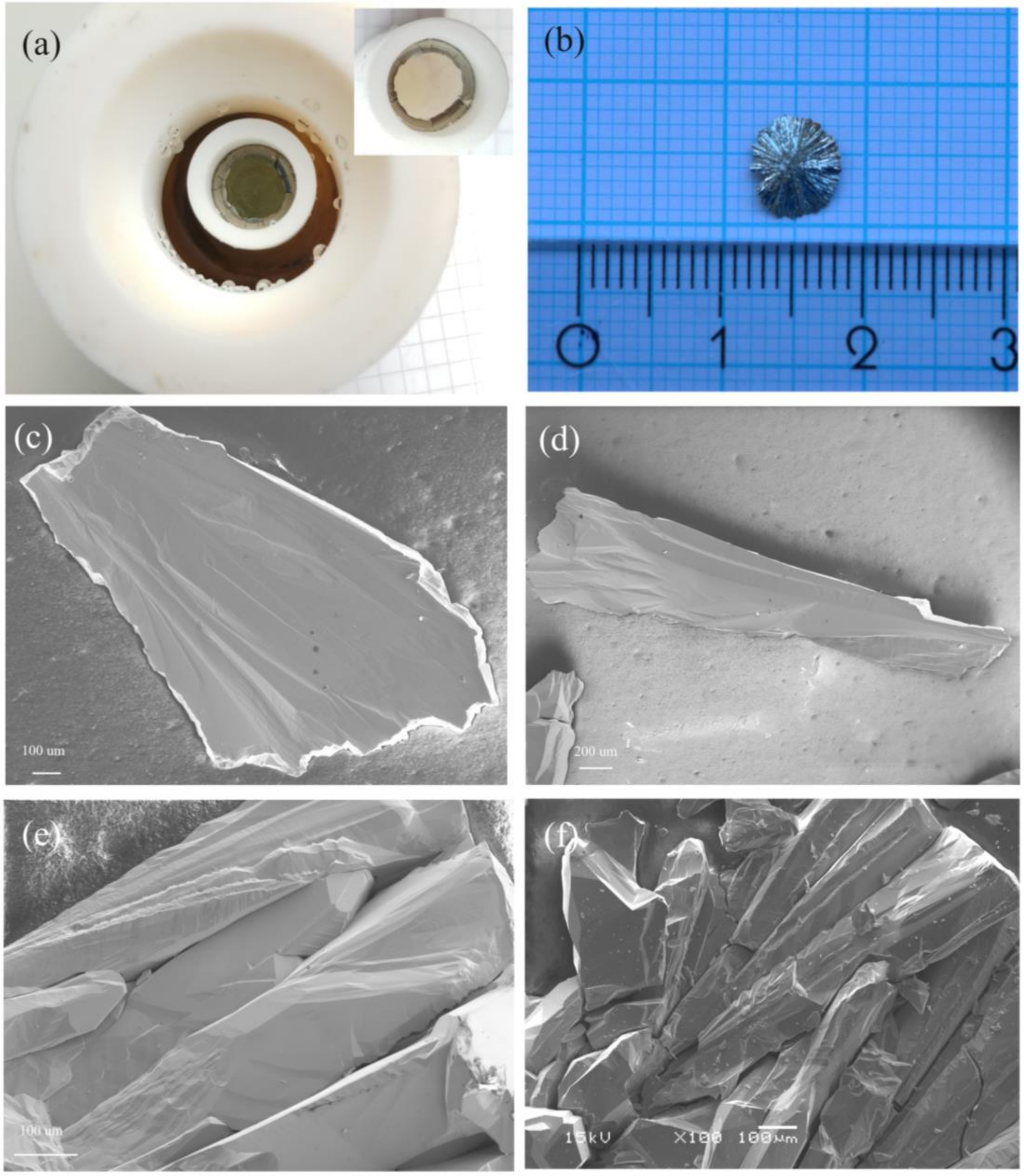

S-Figure3. $FeCl_2$-$Na_2S_2O_3$ space separated reaction system: (a) after reaction the marcasite were formed above the $FeCl_2$ solution in a form of thin palate (180 °C 1 day). (b) large marcasite single can be obtained by using the large size PTFE liner with 14 days at 180 °C. (c-f) the obtained large marcasite has a shiny metallic luster with prismatic macro morphology.

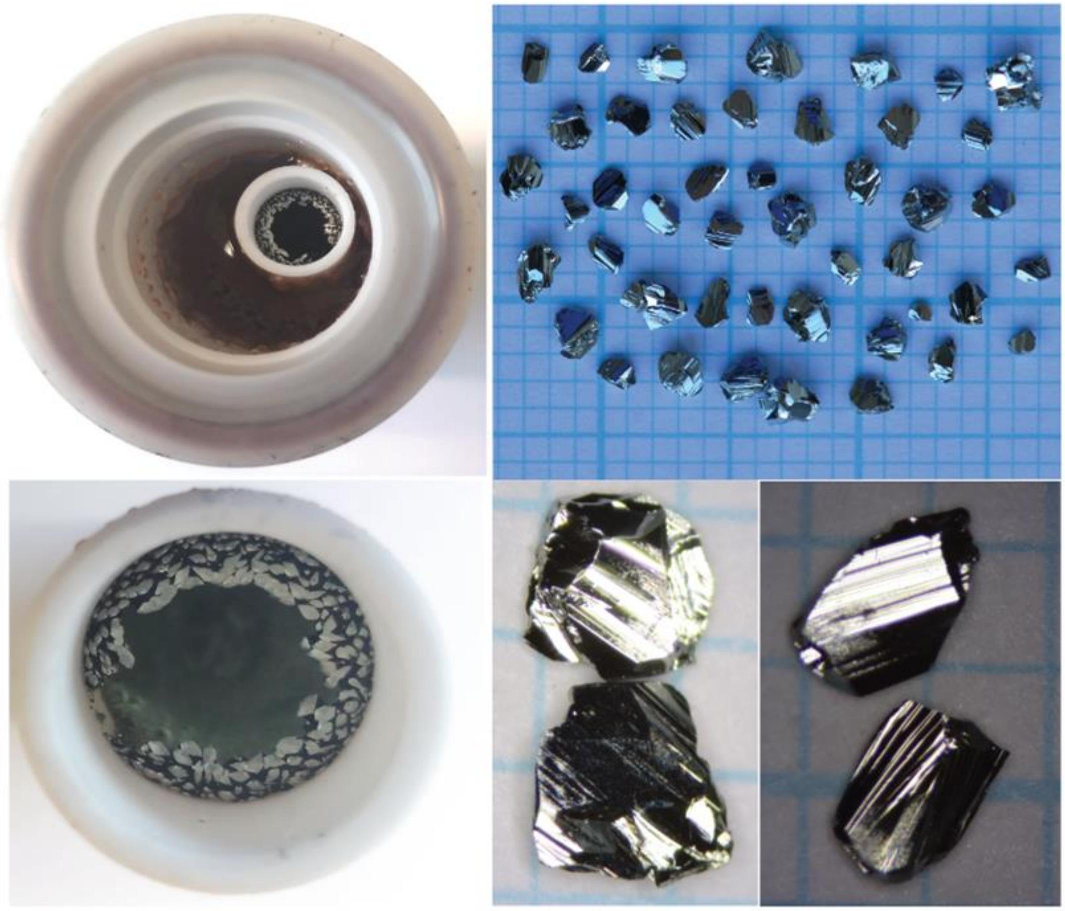

S-Figure 4. $FeCl_2$-$Na_2S_2O_3$ space separated reaction system with large PTFE liner at 240 °C for 3 days, the obtained large marcasite has a shiny metallic luster with some perfect crystal surfaces (above the $FeCl_2$ solution). On the walls of the liner, we can observe some large sulfur particles produced from $Na_2S_2O_3$ solution decomposition. Meanwhile, we can collect some products in the $FeCl_2$ solution.

5. Decomposition of $Na_2S_2O_3$ solutions:

1 mol/L $Na_2S_2O_3$ solution has a pH value of 6.61 at room temperature. At above 165 ˚C, it decomposed to produce H_2S gas and sulfur. After evaporation of the water from the solution, we collect a lot of powders. Pxrd measurement showed diffraction peaks from Na_2SO_4 and Na_2S. Therefore, the decomposition of $Na_2S_2O_3$ solution can be expressed by following reaction:

$$Na_2S_2O_3 + H_2O \longrightarrow H_2S(gas) + Na_2SO_4 + sulfur$$

6. pH value measurement of the $FeCl_2$ solution after reaction of the space separated hydrothermal synthesis:

The used 1M $FeCl_2$ solution in the space separated hydrothermal reaction has a staring pH value of 2.08. In order to see the pH value changes after reaction, we measured the pH values of the $FeCl_2$ solutions after 1 day reaction (in S-Table 4). We found the pH values became very low after 1 day reaction.

S-Table 4. Final compounds pxrd patterns for the space separated hydrothermal reactions and pH values of the $FeCl_2$ solutions after reaction:

Temperature (˚C)	Pyrite (%)	pH value of $FeCl_2$ solution
170	0	1.08
180	0	0.77
190	0	0.62
200	1	0.47
210	3	0.46
220	8	0.45
230	14	0.43
240	25	0.34

7. PXRD pattern of FeS_2 product prepared by non-space-separated hydrothermal reaction.

We have performed some non-space-separated hydrothermal reactions of the 1M $FeCl_2$ and 1 M $Na_2S_2O_2$ solutions with the same process of space-separated reaction to check the final products. We find the obtained products were a mixed phase of FeS_2 marcasite and pyrite with some sulfur impurities. Changing the pH values of the solutions and reaction temperatures did not yield phase pure product.

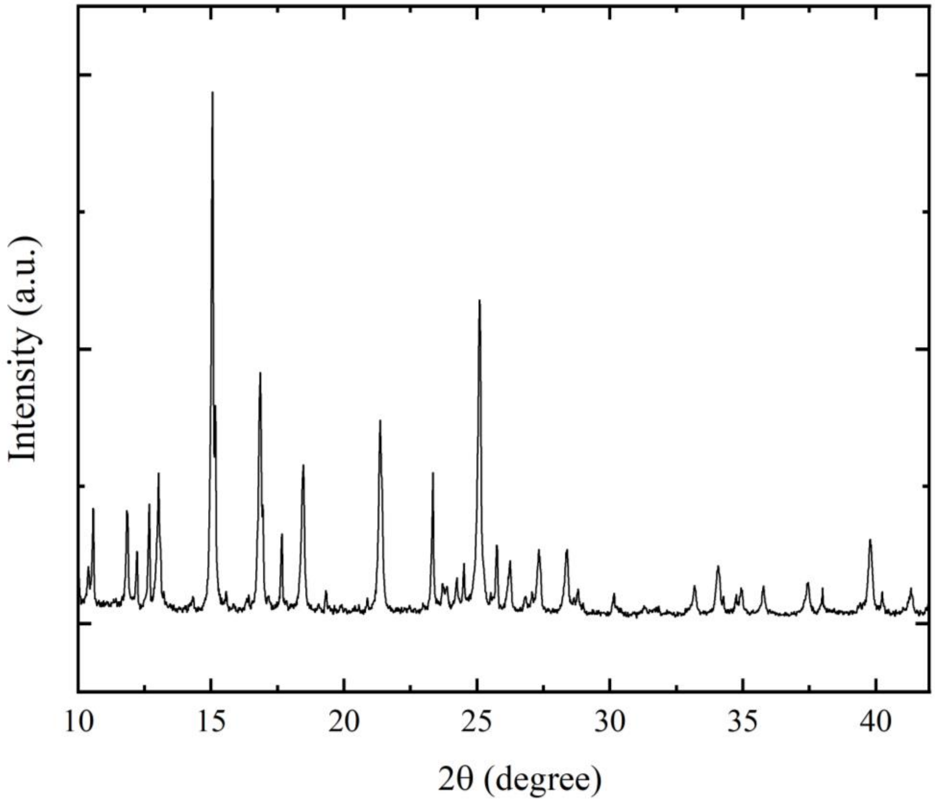

S-Figure 5. pxrd pattern of the FeS_2 products (Mo Kα, at room temperature) from mixed $FeCl_2$ and $Na_2S_2O_3$ solution reaction at 180 ˚C. The products are a mixture of FeS_2 marcasite and pyrite with some sulfur.

8. **EDS analysis of the obtained FeS_2 Marcasite single crystals**

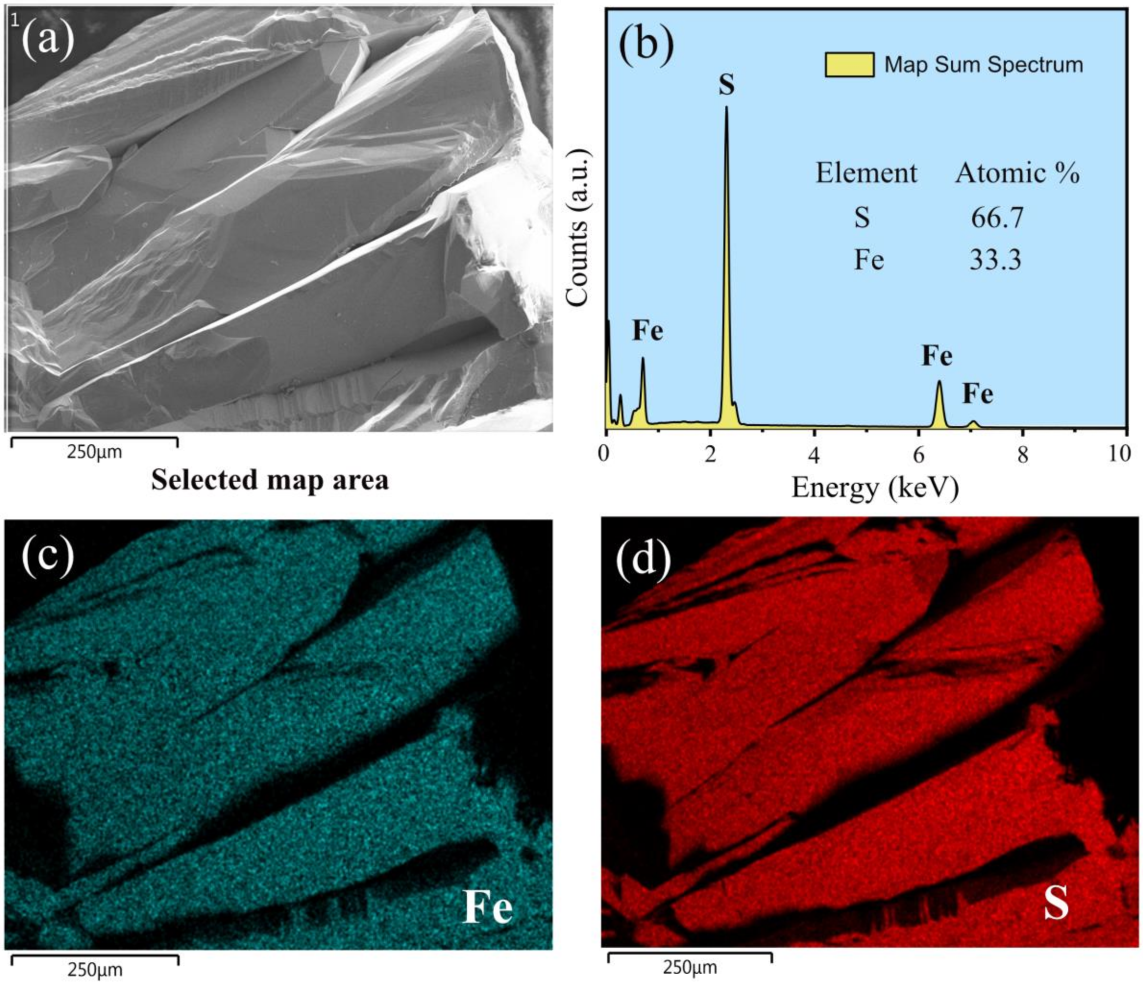

S-Figure 6. EDS analysis of FeS_2 Marcasite single crystals obtained at 180 °C. (a) selected area for the EDS analysis, (b) EDS of the selected area (S/Fe atom ratio is 66.7/33.3), (c) elemental mapping for Fe, and (d) elemental mapping for S.

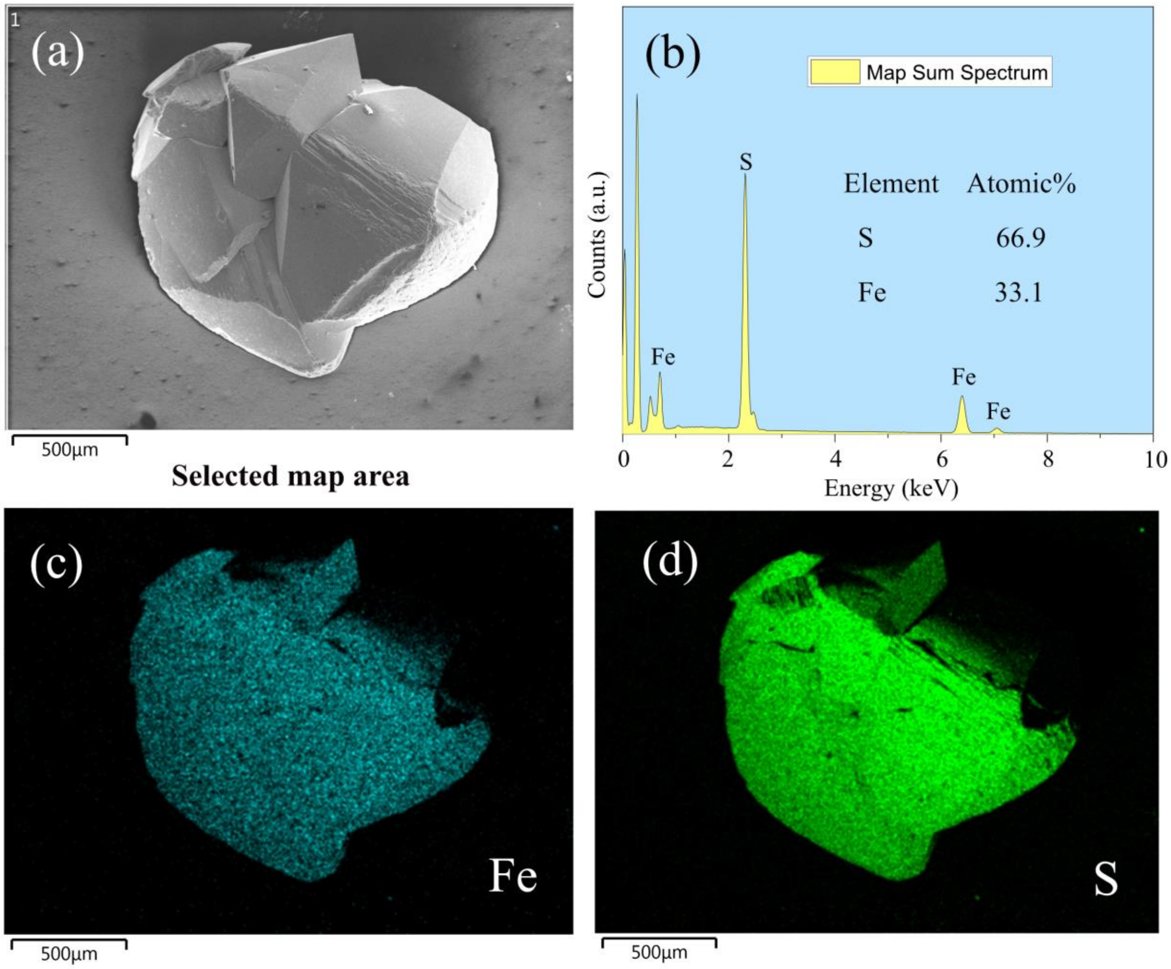

S-Figure 7. EDS analysis of FeS_2 Marcasite single crystals obtained at 240 °C. (a) selected area for the EDS analysis, (b) EDS of the selected area (S/Fe atom ratio is 66.9/33.1), (c) elemental mapping for Fe, and (d) elemental mapping for S.

9. Thermogravimetry test of Marcasite in O_2 atmosphere

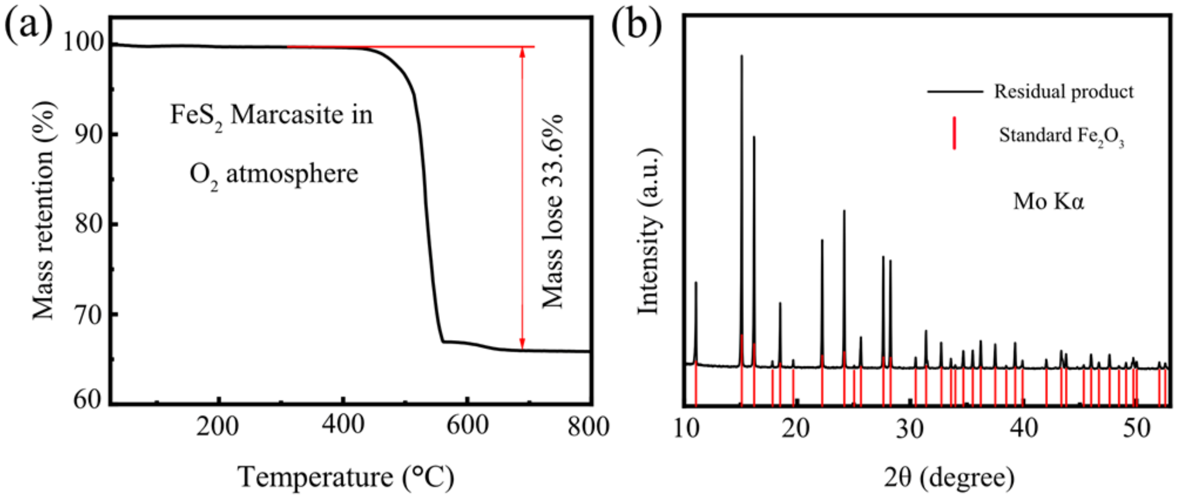

S-Figure 8. (a) thermogravimetry (TG) test of Marcasite single crystals in O_2; (b) pxrd pattern of the residual product from the TG test.

The EDS, which only analysis very limited area of the sample, usually give unreliable elemental composition analysis result. By comparison, the thermogravimetry (TG) test can measure large amounts of samples, and this method is more reliable than the EDS measurement. Here, we used TG measurement to check the chemical composition of FeS_2 Marcasite in O_2 at a temperature range of 25-800 °C. In this process, the S in FeS_2 Marcasite reacted with O_2 to form SO_2, while the Fe formed Fe_2O_3, which can be confirmed by PXRD measurement on the residual products after the TG measurement (figure b). With the mass loss (33.6%) of the test (figure a), we checked the chemical composition of FeS_2 Marcasite according to the following processes:

Initial FeS_2 sample + O_2 = SO_2 + Fe_2O_3

Initial sample mass: 30.863 mg   Final sample mass (Fe_2O_3): 20.493 mg

Fe_2O_3 (2×55.847+3×16=159.694)

Mass of Fe in the initial sample: m_{Fe} = 20.493×2×55.847/159.694 mg=14.334 mg

Mass of S in the initial sample: m_S= 30.863 mg -14.334 mg = 16.529 mg

Atom ratio of Fe/S in the sample (Fe:55.847; S:32.066):

(14.334÷55.847) / (16.529÷32.066) = 0.257/0.515 = 0.499

Here, we find the resulted Fe/S atom ratio in our FeS_2 Marcasite samples is in perfect agreement with the ideal ratio.

10. Preparation of Pyrite single crystals from Marcasite single crystal

The obtained large marcasite single crystals were sealed in a silica tube with 1/3 atm argon and heated in a furnace for 1 day at 450 °C. We performed single crystal measurement on the obtained pyrite single crystals (S-Table 5). We find the obtained pyrite single crystals have no difference with those prepared by other method or the natural pyrite.

S-Table 5 Crystal data and structure refinement for FeS_2 Pyrite single crystal

Empirical formula	FeS_2
Formula weight	119.97
Temperature/K	160(1)
Crystal system	cubic
Space group	Pa-3
a/Å	5.41027(15)
b/Å	5.41027(15)
c/Å	5.41027(15)
α/°	90
β/°	90
γ/°	90
Volume/Å^3	158.364(13)
Z	4
ρ_{calc}g/cm^3	5.032
μ/mm^{-1}	95.830
F(000)	232.0
Crystal size/mm^3	0.05 × 0.04 × 0.02
Radiation	CuKα (λ = 1.54184)
2Θ range for data collection/°	28.576 to 145.828
Index ranges	$-5 \leq h \leq 6, -6 \leq k \leq 5, -6 \leq l \leq 6$
Reflections collected	509
Independent reflections	57 [R_{int} = 0.0374, R_{sigma} = 0.0170]
Data/restraints/parameters	57/0/7
Goodness-of-fit on F^2	1.320
Final R indexes [I>=2σ (I)]	R_1 = 0.0183, wR_2 = 0.0444
Final R indexes [all data]	R_1 = 0.0183, wR_2 = 0.0444
Largest diff. peak/hole / e Å^{-3}	0.39/-0.34

11. Raman spectra of FeS_2 Marcasite and Pyrite single crystals

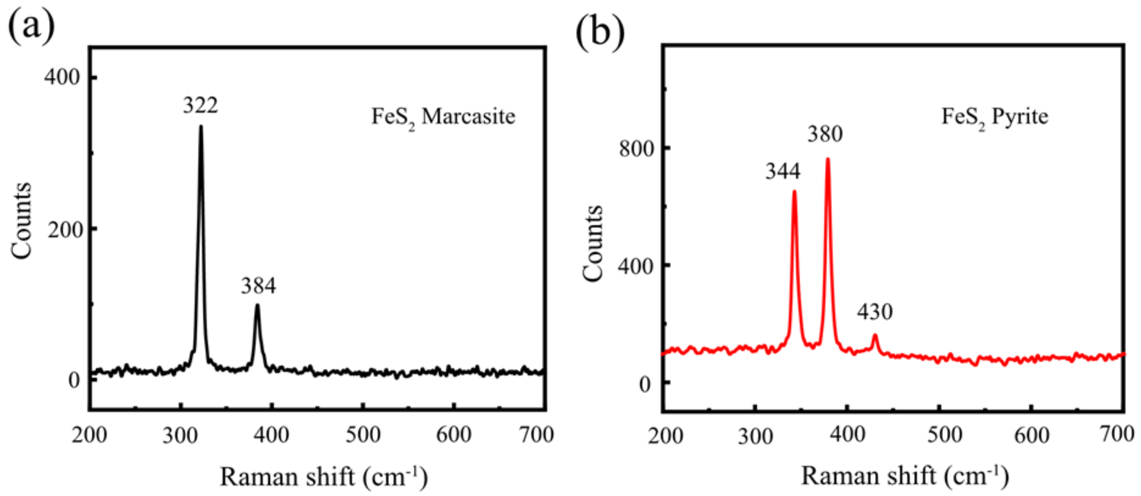

S-Figure 9. Raman spectra of FeS_2 Marcasite (a) and Pyrite single crystals (b)

12. The optical band gap measurement of Marcasite and Pyrite:

The optical band gap measurement of Marcasite and Pyrite by means of UV-Vis-NIR diffuse reflectance spectrum in the region of 200-2000 nm.

The following relational expression proposed by Tauc is used:

$$(h\nu\alpha)^{n} = A(h\nu - E_g)$$

Here, h is the Planck constant, ν is frequency, α is the absorption coefficient, E_g is band gap and A is a proportional constant. The value of the exponent n denotes the nature of the sample transition: n=2 corresponds to direct allowed transition, and n=0.5 corresponds to indirect allowed transition. In our UV-Vis-NIR diffuse reflectance spectrum measurement, the acquired diffuse reflectance spectrum is converted to Kubelka-Munk function:

$$F(R) = (1-R)^2/2R$$

Thus, the vertical axis is converted to quantity F(R), which is proportional to the absorption coefficient α. The α in the Tauc equation is substituted with F(R), and the above Tauc equation becomes:

$$(h\nu F(R))^{n} = A(h\nu - E_g)$$

We plotted (hν - $(h\nu F(R))^2$) and (hν - $(h\nu F(R))^{0.5}$) curves for Marcasite and Pyrite. A line is drawn tangent to the point of inflection on the curve, and the hν value at the point of intersection of the tangent line and the horizontal axis is the band gap E_g value.

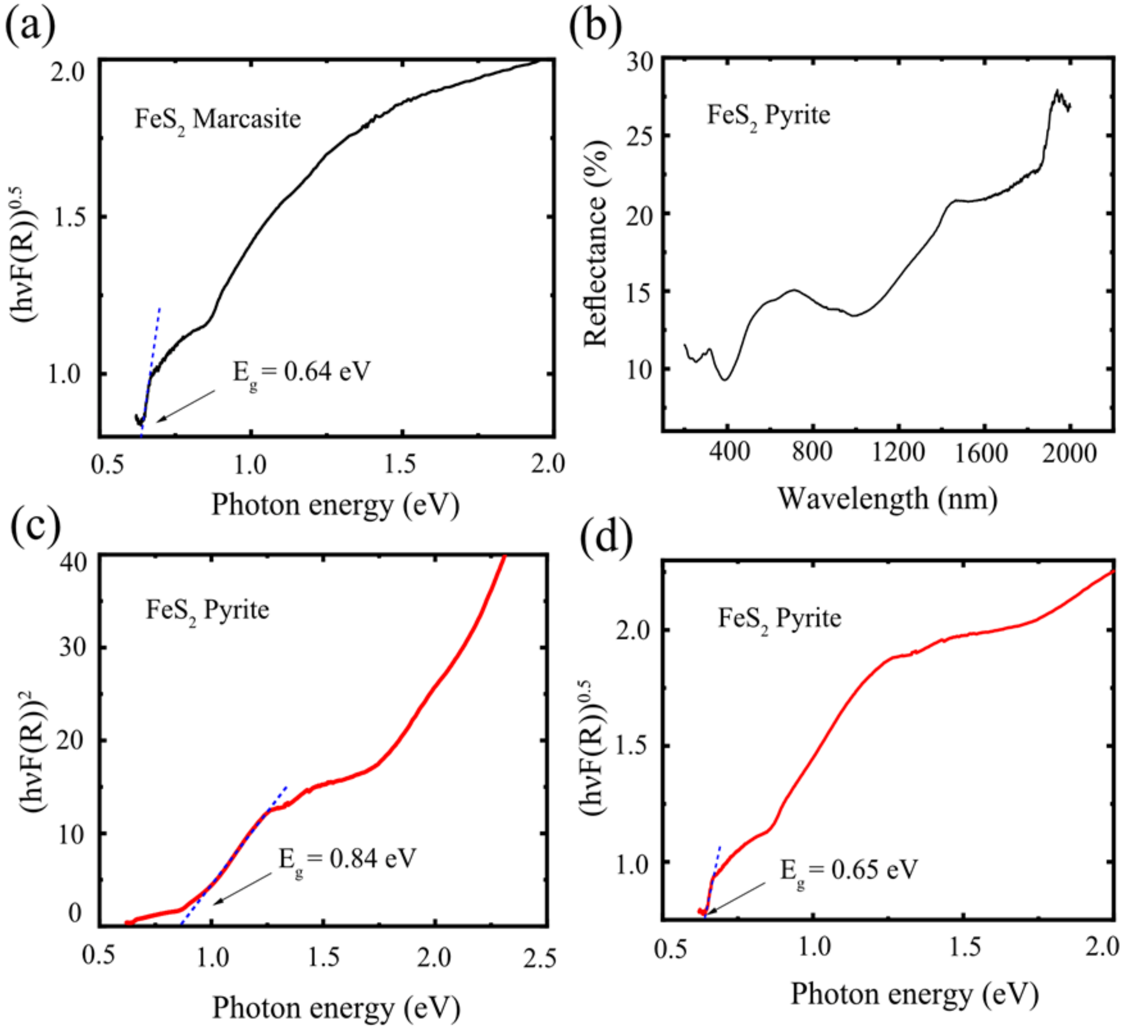

S-Figure 10. Optical band gap determination of FeS_2 Marcasite and Pyrite single crystals from the UV-Vis-NIR diffuse reflectance spectrum.

13. Calculation of electrical resistance band gap and Hall coefficient

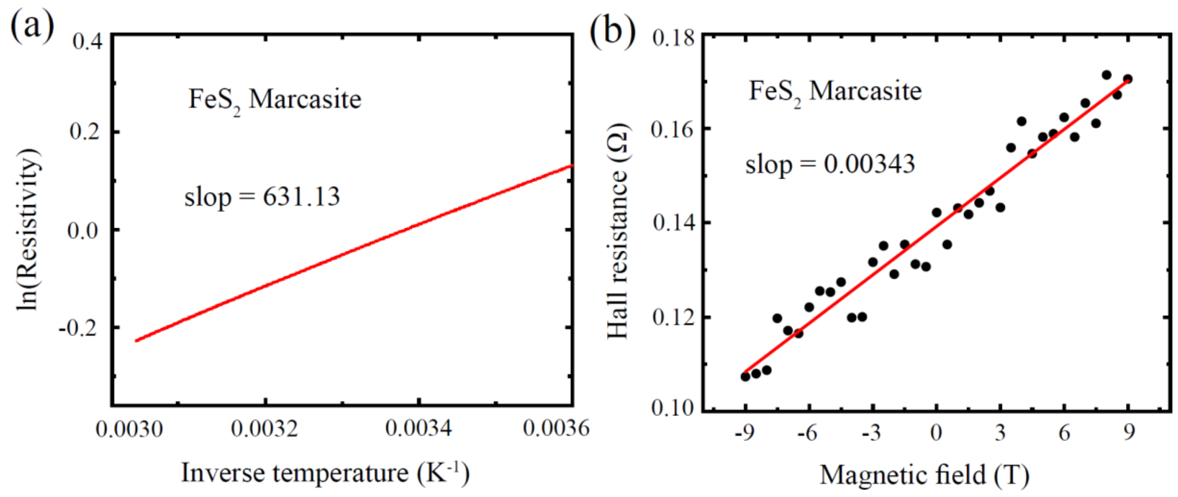

S-Figure 11. (a) electrical resistance band gap fitting of Marcasite; (b) Hall coefficient determination.

The electrical resistance band gap is calculated as following:

$$\rho = \rho_0 \exp\left(\frac{E_a}{2k_B T}\right)$$

Where ρ_0 is a pre-exponential constant, $k_B = 8.617 \times 10^{-5}$ eV K^{-1} is the Boltzmann constant, and E_a is the activation energy.

$$\ln\rho = \rho_0 + \frac{E_a}{k_B T}$$

The slop of 1/T – lnρ (631.13 K) corresponds to $E_a/2k_B$.

Ea = 2 ×631.13 × 8.617 × 10^{-5} eV = 0.11 eV

Calculation of Hall coefficient:

The Hall coefficient R_H is calculated according to:

$$R_H = \frac{V \times t}{I \times B}$$

Here, V [V] is the Hall voltage, t [m] is the thickness of the conductor, I [A] is the current running through the conductor, B [T] is the magnetic field. The thickness of our measured sample is (0.44 mm). The Hall coefficient R_H for Marcasite is $R_H = 3.43 \times 10^{-3} \times 4.4 \times 10^{-4}$ $m^3/C = 1.51 \times 10^{-6}$ m^3/C.

14. XES spectrum of FeS_2 Marcasite and Pyrite measured on the S-L edge

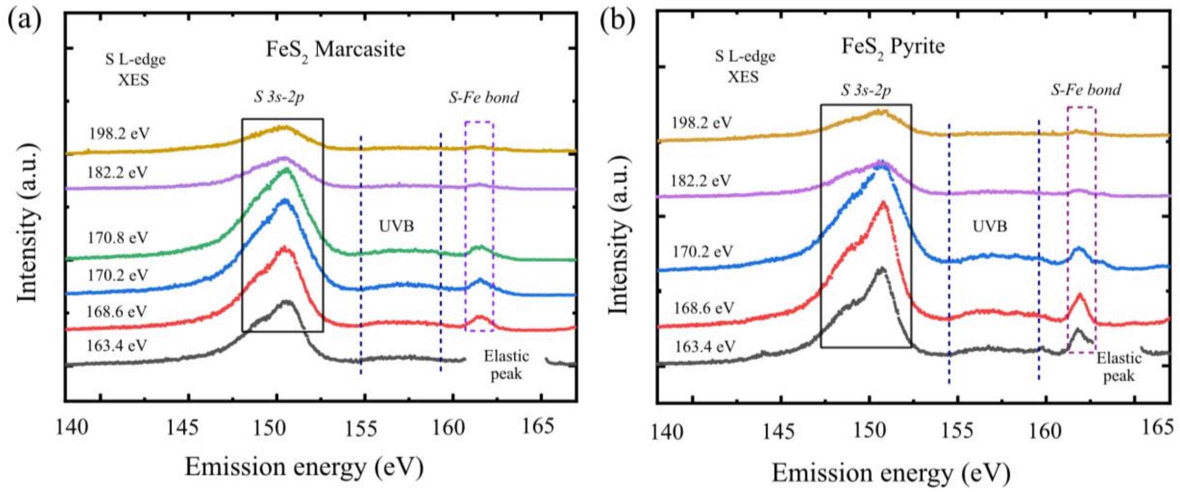

S-Figure 12. X-ray emission spectra of S L-edges with the excitation energy indicated by the indications for FeS_2 Marcasite (a) and Pyrite (b). (The very strong elastic peaks at around 163 eV in the XES spectrums excited with 163.4 eV photon energy were removed for a clear comparison.)

15. Band gap Determination process of FeS_2 Marcasite and Pyrite based on the XAS and XES measurement on S-L edge:

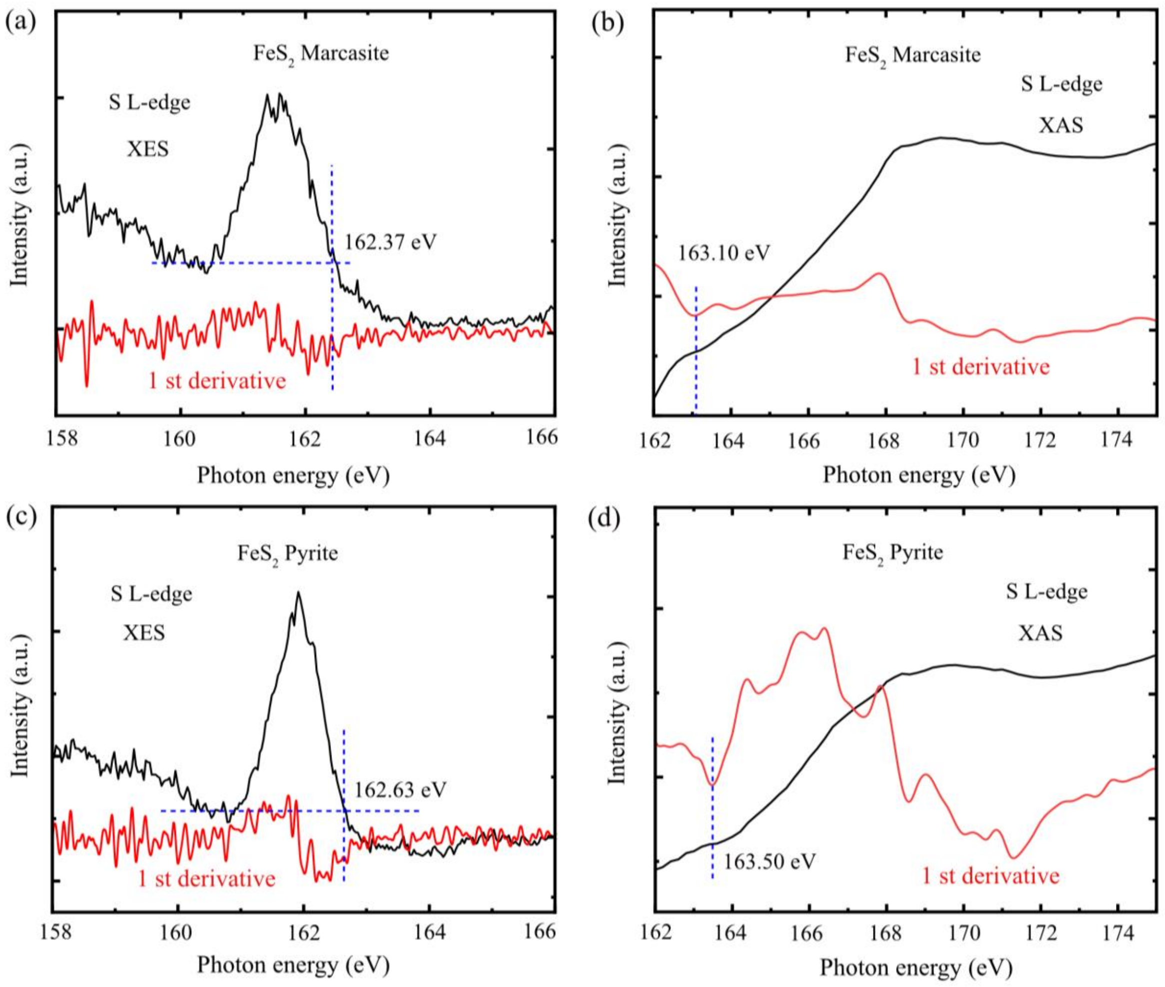

S-Figure 13. Threshold values of valence-band maximum and conduction-band minimum determination for the Marcasite and Pyrite.

References:

[1] Rigaku Oxford Diffraction, **2015**.
[2] Clark, R. C.; Reid, J. S. *Acta Cryst. A* **1995**, 51, 887.
[3] *CrysAlisPro* (version 1.171.39.13a), Rigaku Oxford Diffraction, **2016**.
[4] "*A complete structure solution, refinement and analysis program*" - Dolomanov, O. V.; Bourhis, L. J.; Gildea, R. J.; Howard, J. A. K.; Puschmann, H. *J. Appl. Cryst.* **2009**, 42, 339.
[5] "*SHELXT - Integrated space-group and crystal-structure determination*" - Sheldrick, G. M. *Acta Cryst. A* **2015**, 71, 3.
[6] "*Crystal structure refinement with SHELXL*" - Sheldrick, G. M. *Acta Cryst. C* **2015**, 71, 3.
[7]"*Structure validation in chemical crystallography*" - Spek, A. L. *Acta Cryst. D* **2009**, *65*, 148.
